# Three Sugical Cases

**Published:** 1855-09

**Authors:** J. C. Nott

**Affiliations:** Mobile, Alabama


*Three Surgical Cases.* Reported by J. C. NOTT, M. D., of Mobile, Alabama.

The following cases possess sufficient novelty and practical value to deserve publication:

CASE I.—*Spina Bifida cured by Excision.*—The subject of this case, aged one month, was a male, and the child of an Irish woman; it presented, about the middle lumbar vertebra, a tumor an inch and a half in diameter, nearly circular, and elevated about three-quarters of an inch. The appearance of the tumor was unusual, and the first impression on my mind was that of a fungus hematodes; the summit was nearly flat, of a reddish chocolate color, and in the centre was a thin pellucid membrane of about three-fourths of an inch in extent, through which could be seen serous fluid. The most graphic description I can give of the appearance of the tumor is that it resembled a half-ripe carbuncle with a Malaga grape buried in the centre; had it not been for this deficiency of skin in the centre I should have been much embarrassed to form a diagnosis. Guided by a case somewhat similar, though smaller, reported by Dr. Mott, of New York, in his Appendix to Velpeau, I determined to extirpate the entire mass. I accordingly, on the 15th of March, 1855, in the presence of Drs. J. Hamilton, Vetchum, and Anderson, and my student, Mr. Childs, inclosed the tumor by two elliptical incisions in the direction of the spine, and dissected it out completely; the tumor was found to consist simply of skin, cellular tissue, and the membranes of the spine distended with serum. After the sac was removed, an opening into the spinal canal was exposed about the size of the end of the finger, and a tablespoonful of fluid escaped.

It was then dressed by bringing the edges together by a single pin and twisted suture, and placing above and below strips of the adhesive plaster.

The dressing was removed on the third day, and complete adhesion had taken place by first intention, except the portion included between the pin and ligature, which sloughed; this left a narrow gaping ulcer immediately over the opening in the spine, and I felt some apprehension about the result. I did not reflect on the extreme vascularity and tenderness of the skin of a child a month old, and put too much stress on a single point; it would have been more scientific to have made a longitudinal cut on each side to free the skin, and to have used two pins instead of one. The case, however, did well; granulations were thrown out, and the ulcer soon closed, and at the end of two weeks the healing was perfect and the parts firm and solid.

The child had no constitutional disturbance whatever; slept and nursed as usual. Two months have now passed, and the cure seems to be complete.

CASE II.—*Testicle Containing Hair—Removal*—The subject of this case, aged 22 years, was a patient of Dr. F. A. Ross, who called me in consultation. Says the right testicle was larger than the other in childhood, but never pained until the last five years, since which time it has been enlarging, and has given a good deal of pain. On examination, a tumor was found larger than the first, about half of which was hydrocele and the remainder enlargement of the testicle; pus had formed and pointed at the epididymis, but no opening had ever occurred.

It was determined to remove the testicle and sac together, which was done. The dissection of the mass after removal revealed the only points of special interest. The tunica vaginalis was much thickened, and contained a gill of limpid fluid. The testicle itself was enlarged to about four times its normal size. On opening it, the tunica albuginea was found a good deal thickened, and the glandular contents, if any ever existed, entirely destroyed by suppuration; the tunica albuginea was a complete sac, entirely filled with thick pus, about the consistence of boiled custard, mixed up with hair, from half an inch to four inches long. I sent specimens of the hair to Peter A. Brown, esq. and Prof. Leidy, of Philadelphia, but they could detect nothing on examination with the microscope peculiar in the hair, worthy of remark.

It is more than probable that this development of hair was congenital, and I have no speculations to offer on the subject. We have many instances of development of hair in cysts in various parts of the body; still this case is interesting both to the physiologist and pathologist, as well as to the surgeon.

CASE III.—*Necrosis of Lower Jaw.*—The subject of this case was a mulatto boy belonging to Major Haden, of Selma, Alabama, about eight years old. He had been kicked by a horse on the lower jaw six months before he was brought to me in Mobile, but I could not obtain any satisfactory history of the case. The jaw seemed to have been fractured, and when I first saw him there existed enormous swelling, with suppuration, and two openings, one at the angle of the jaw and the other near the chin On passing a probe from one opening to the other dead bone was detected. It was determined to make a free opening with the view of removing the diseased bone, but the extent of the operation could not be determined beforehand. A free opening was made externally down to the bone, from the angle of the jaw along its base to the chin; and, on introducing the finger, the body and ramus of the bone were found extensively denuded and bathed in pus. The base of the bone was enlarged in thickness to about t' ree times its natural dimensions; and, on passing the finger along the outer surface of the bone from the ramus forward towards the chin, a deep suture was felt along the shaft of the bone; this sulcus seemed to be the line between the living and dead bone. The diseased bone was not yet separated at its anterior extremity, but was still firmly adhering to the sound bone at the chin. I dissected loose the soft parts freely, and divided the bone at its anterior part with strong bone nippers; the ligamentous attachments of the condyloid and coronoid processes being destroyed by ulceration, I had little difficulty in extracting the bone with a pair of large forceps; the whole of the ramus and lower half of the body of the bone as far forward as the canine tooth was all brought away in a solid piece; the teeth, alveolar processes, and upper margin of bone were all left *in situ.* The boy had none but his twenty milk teeth, and the interesting point of the case was to see whether the teeth and portion of bone left behind could still be nourished and remain healthy after so large a piece of bone was removed, *including the nutritious artery.* The case did well, and was soon sent home to the country.

I received a letter a few days ago from Dr. Wm. P. Reese, from which I give the following extract, dated about two months and a half after the operation:

"I have this day examined the little boy William, of Major Haden; he is quite well with the exception of a very small half closed fistulous opening at the anterior terminus of the cicatrix, from which there is very slight exudation; the teeth, gums, and alveoli are *in situ,* and apparently perfectly healthy; his general health is good."

As I had to make an artificial division of the bone at the chin, I expected slow healing then, and probably the discharge of some small fragments of carious bone.

This case will be a curious one to watch, both in a pathological and physiological point of view. Will such a section of bone be able to sustain itself and the inclosed teeth? will new teeth replace the old ones when cast off? will this fragment of jaw have sufficient strength for mastication? I hope to be able to keep a watch upon the case through my friend, Dr. Reese.—*American Journal Medical Sciences.*

